# Inertial Measures of Motion for Clinical Biomechanics: Comparative Assessment of Accuracy under Controlled Conditions - Effect of Velocity

**DOI:** 10.1371/journal.pone.0079945

**Published:** 2013-11-19

**Authors:** Karina Lebel, Patrick Boissy, Mathieu Hamel, Christian Duval

**Affiliations:** 1 Faculty Of Medicine And Health Sciences, Orthopedic Service, Department Of Surgery, Université De Sherbrooke, Sherbrooke, Quebec, Canada; 2 Research Center On Aging, Sherbrooke, Quebec, Canada; 3 Interdisciplinary Institute For Technological Innovation (3IT), Université De Sherbrooke, Sherbrooke, Quebec, Canada; 4 Department Of Kinesiology, Université Du Québec À Montréal, Montreal, Quebec, Canada; 5 Centre De Recherche Institut Universitaire De Gériatrie De Montréal, Montreal, Quebec, Canada; University of Utah, United States of America

## Abstract

**Background:**

Inertial measurement of motion with Attitude and Heading Reference Systems (AHRS) is emerging as an alternative to 3D motion capture systems in biomechanics. The objectives of this study are: 1) to describe the absolute and relative accuracy of multiple units of commercially available AHRS under various types of motion; and 2) to evaluate the effect of motion velocity on the accuracy of these measurements.

**Methods:**

The criterion validity of accuracy was established under controlled conditions using an instrumented Gimbal table. AHRS modules were carefully attached to the center plate of the Gimbal table and put through experimental static and dynamic conditions. Static and absolute accuracy was assessed by comparing the AHRS orientation measurement to those obtained using an optical gold standard. Relative accuracy was assessed by measuring the variation in relative orientation between modules during trials.

**Findings:**

Evaluated AHRS systems demonstrated good absolute static accuracy (mean error < 0.5^o^) and clinically acceptable absolute accuracy under condition of slow motions (mean error between 0.5^o^ and 3.1^o^). In slow motions, relative accuracy varied from 2^o^ to 7^o^ depending on the type of AHRS and the type of rotation. Absolute and relative accuracy were significantly affected (p<0.05) by velocity during sustained motions. The extent of that effect varied across AHRS.

**Interpretation:**

Absolute and relative accuracy of AHRS are affected by environmental magnetic perturbations and conditions of motions. Relative accuracy of AHRS is mostly affected by the ability of all modules to locate the same global reference coordinate system at all time.

**Conclusions:**

Existing AHRS systems can be considered for use in clinical biomechanics under constrained conditions of use. While their individual capacity to track absolute motion is relatively consistent, the use of multiple AHRS modules to compute relative motion between rigid bodies needs to be optimized according to the conditions of operation.

## Introduction

Mobility is a fundamental part of self-care activities and instrumental activities of daily living within an individual's place of residence or the community. It is achieved through coordinated physiological and mechanical interactions between bones, muscles, ligaments and joints under the control of the central and peripheral nervous systems. With aging and disease, balance, strength, joint health, motor coordination and cognitive processing can be affected and introduce mobility impairments. Mobility impairments can take many forms (difficulty in kneeling, sitting down, rising, standing, walking, and/or climbing stairs) that have functional impacts in everyday life.

The evaluation of mobility impairments is the key to many clinical practices in the field of orthopaedics, neurology, geriatrics and rehabilitation. From the determination of the appropriate intervention to assessing changes related to this intervention, outcomes related to the measurement of mobility impairments are used across the continuum of care. Traditionally, mobility impairments are measured using self-report questionnaires, performance based clinical tests or with instrumented techniques such as 3D capture of joint motion with optical or magnetic tracking systems. 3D capture of joint motion with optical or magnetic tracking systems is expensive, complex to configure and operate for clinicians but offer highly accurate tracking within a given volume [1]. Accurate tracking is however limited to a specific motion capture volume with either a clear line of sight between multiples cameras and the markers used for the optical systems, or without any ferrous elements to minimize magnetic tracker distortion for the magnetic tracking systems. Furthermore, the motion capture volume is generally constrained in space and the equipment (camera, transmitter and receiver) has to be positioned optimally in the environment to obtain this accuracy.

In the field of biomechanics, inertial measurements of motion [2] is emerging as an alternative to optical or magnetic 3D motion capture systems for the measurement of mobility impairments [3]–[6]. The inertial measurement of motion relies upon the determination of an absolute orientation expressed in a global coordinate system, based on gravity and magnetic North. To do so, such systems make use of data from inertial sensors (accelerometers, gyroscopes and magnetometers) which, combined with a fusion algorithm (e.g. Kalman filter), allows the determination of the global orientation of the module. The merge of three types of inertial sensors with a fusion algorithm is referred to as Attitude and Heading Reference Systems (AHRS). By attaching an AHRS module on a limb, one can determine the orientation of that limb in a global reference. Analysis of orientation variations can, for example, be used to analyse trunk kinematics [7]. If two limbs have AHRS modules attached to them, it is then possible to reconstruct the kinematics of joint motion for a specific joint. Ferrari et al. [5] used multiple AHRS to measure gait parameters, mainly hip, knee and ankle joint angles during normal walk while Cutti et al. [4] developed a protocol that measures the scapulothoracic, humerothoracic and elbow 3D kinematics, again using AHRS. One of the main interests of using AHRS in biomechanics is that it allows functional evaluation of motion in realistic environments and conditions with fewer operational constraints then optical or magnetic motion capture systems. Their long-term recording capabilities also allow the capture of changes and variability in motion in a given scenario (e.g. sustained walking with turning, stair ascend and descend over one floor, etc.).

While AHRS offer advantages over traditional methods of motion capture for use in clinical biomechanics, they also have limitations, which haven’t been extensively documented. The accuracy specifications provided by the manufacturers are presented with caveats and are undocumented, which raises a lot of questions. Are all the commercially available systems comparable? Can someone use these systems out of the box and trust the measurements? How are the measurements truly affected by environmental variations and specific motion?

Recent studies have explored the validity of AHRS under different contexts of use in biomechanics [4], [5], [7]–[14]. Picerno et al. [11] addressed the accuracy of AHRS by evaluating the consistency of multiple modules in determining their orientation with respect to a common and invariant global frame, using 9 IMUs aligned and fixed on a Plexiglas plank. The authors concluded that the IMUs tested defined their orientation differently, with a worst-case discrepancy measured of 5.7° under different static conditions. Cutti et al. [13] again used a rigid plate with four modules. A series of static and dynamic acquisitions were performed, and orientation errors for each pair of modules were computed. The effect of velocity and direction of rotation on precision was then assessed, revealing a worst-case orientation error of 5.4° and 11.6° for mean rotation velocities of 180°/s and 360°/s respectively. Brennan et al [12] used an instrumented Gimbal modelling of a right knee to assess accuracy of a pair of IMU. Comparing the inertial measurement with a potentiometer gold standard, they identified root-mean-square errors of 3.2° for flexion/extension, 3.4° for abduction/adduction and 2.9° for internal/external rotation. Although this setup considered the IMUs in their context of use and that the Gimbal was built to minimize alignment errors, it is again based on manually-controlled, un-reproducible conditions and does not allow differentiating between absolute and relative errors, i.e. single module measurement error versus global reference discrepancies.

Most of those validity studies were performed on a single type of system at a time, in so-called ‘clean’ environments, and procedures varied from one study to the other, making it very hard to compare the different conclusions. The scope of the present study is therefore to characterize the criterion validity of different types of market-available AHRS using a controlled bench test with an optical motion analysis system as gold standard. Specifically, the objectives of this paper are: 1) to describe and to compare the absolute and relative accuracy of multiple units of three different types of AHRS systems under various types of motion; and 2) to evaluate the effect of motion velocity on the accuracy of these measurements.

## Materials and Methods

### Attitude and Heading Reference Systems (AHRS)

**Figure 1:**
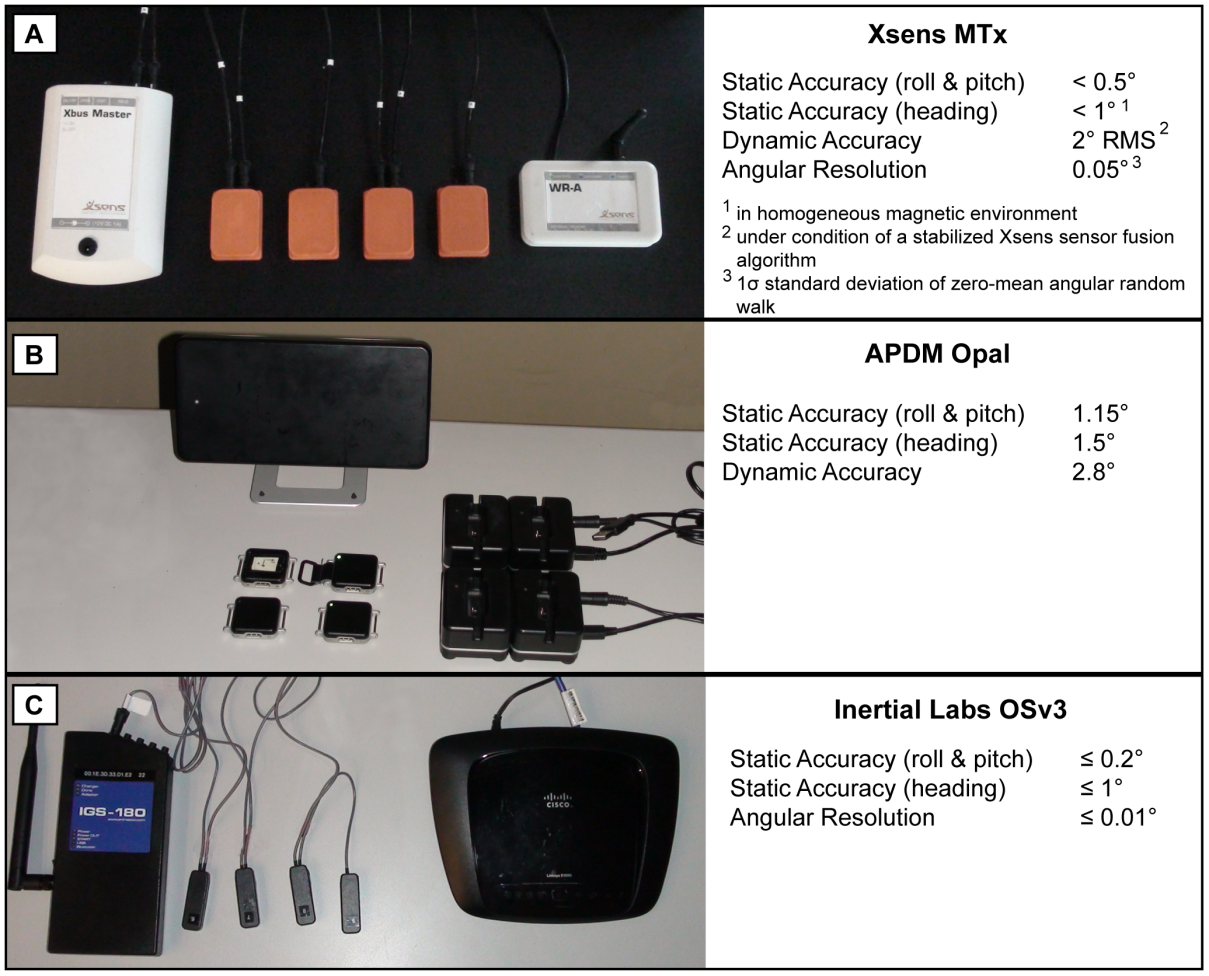
Selected Systems (A) MTx from Xsens (B) APDM Opal and (C) OSv3 from Inertial Labs. AHRS Systems and their Advertised Technical Specifications: (A) Xsens MTx, (B) APDM Opal, and (C) Inertial Labs OSv3.

The development of AHR systems has been growing rapidly in the last three years in terms of research publications and commercialization of new systems and algorithms. The current study considers three market available AHRS systems for biomechanics from established companies (Xsens [15], Inertial Lab [16], APDM [17]). The selected systems all integrate 3-D accelerometers, 3-D gyroscopes and 3-D magnetometers within each sensing unit or module as well as a fusion algorithm allowing orientation data to be computed from the sensors measurements. Figure 1 illustrates the different AHRS systems and the accuracy of the orientation data under different conditions of use for each AHRS system, as marketed by the manufacturers. The selected AHRS systems were purchased independently, at market price, in the last three years by the researchers as part of on-going research projects. Prior to testing, each manufacturer was contacted with a list of the material to be used, the detailed configuration selected, the firmware and software information and the procedure established for their system in order to make sure the results obtained reflect reality and best use of their system. Later, a subset of the data was sent to each company to ensure proper tuning properties were selected for the Gimbal use case scenario (see Bench Test Apparatus below).

The first system considered is the MTx from Xsens [15]. MTx modules, as shown in Figure 1 (A), connect to an Xbus Kit, which communicates wirelessly to a receiver linked to a PC. Data acquisition was performed at 100 Hz using MT Manager Version 1.7.0 configured for human motion, as suggested by the manufacturer. The second system is the Opal modules from APDM [17]. The OPAL modules are wireless and communicate through an access point connected to a PC. Part (B) of Figure 1 shows the OPAL modules and their peripheral equipment. Data acquisition was performed at 128 Hz with Motion Studio v. 1.0.0.201204181627. Motion studio did not have at the time specific settings to adjust for the environment or motion recorded. The third system to be characterized is the OSv3 from Inertial Labs [16]. The specific modules used were initially incorporated within a motion capture suit, the IGS-180 commercialized by Animazoo [18]. For the purpose of the study, a branch of four OSv3 modules was extracted from the suit. Those modules were used for the characterization study along with a MPU in which the different branches of sensors connect. Figure 1 (C) illustrates the equipment used for this part of the study. Data was acquired using Animaserver version 10.4 at 60 Hz and later reprocessed following an update of the SDK library (v. 2.0.1.4703). This updated SDK library came with a list of scenarios from which to choose from in order to configure the fusion algorithm. Upon recommendation from the manufacturer, human motion scenario was selected.

### Bench Test Apparatus

**Figure 2:**
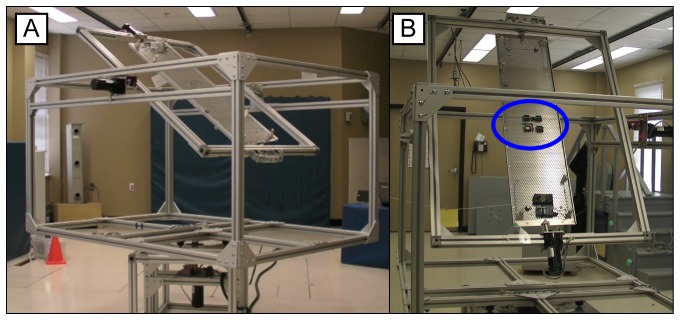
Bench Test Apparatus and Optical Marker Setup. (A) General Bench Test Overview (B) Payload.

In order to enable for AHRS comparison, the experimental setup considered controlled motion (speed and direction) in a standardized tests scenario. The validity criterion of the accuracy of the different AHRS was therefore established independently under controlled conditions using an instrumented bench test (Figure 2). The bench test is comprised of a 3-axes Gimbal table which allows single or multi- axes trajectories of motions for a payload attached to the center plate. Motion is commanded in velocity and is limited to 360°/s per axis. The table is entirely made of aluminum and was designed to minimize the impact of electromagnetic fields induced by motors. The Gimbal table was positioned within a typical clinical biomechanical environment (i.e. gait lab of the Research Centre on Aging, at the CSSS-IUGS hospital). While the space is not optimized for electromagnetic field variations, a minimum clearance of 2 m was established between the center of the Gimbal table and any ferrous material (cabinets, beam…) [19], except for the ferrous material comprised within the Gimbal table motors which was located a minimum of 0.5 m away. The impact of both the permanent magnets of the motors and the magnetic field induced with the motor powered-on were verified experimentally, and were shown to be within magnetometers’ noise level at 0.4 m.

### Experimental Protocol

Each system was evaluated separately on the Gimbal table, using a standardized protocol. Specifically, four modules from the same company were carefully attached to the center plate of the Gimbal table and visually aligned as shown in Figure 2. For each system, a warm-up period in which modules were rotated in all directions, at slow speed, was performed prior to official data collection in order to warm-up the electronics. Experimental conditions included static positions conditions, 1-axis trajectories (slow and fast) as well as 3-axes trajectories (slow and fast) dynamic conditions. Slow and fast velocities were fixed to about 90^o^/s and 180^o^/s. Static positions conditions were recorded in two stages (three prior to the dynamic trials sequence and three afterwards). At each position, a 20s stabilization period was granted to ensure that the dynamic part of the movement did not interfere with the measurement. Mean orientation was computed over the following 30s. For each dynamic condition, three trials were performed. The start-up position of the AHRS during dynamic trials was standardized so to minimize possible effect of inertia on the trials’ repeatability. Furthermore, dynamic trials all considered an initial 5s stabilization period followed by a 2 minutes dynamic motion at commanded speed. The effect of velocity on accuracy was evaluated using the first dynamic 30s of the appropriate trials.

**Figure 3:**
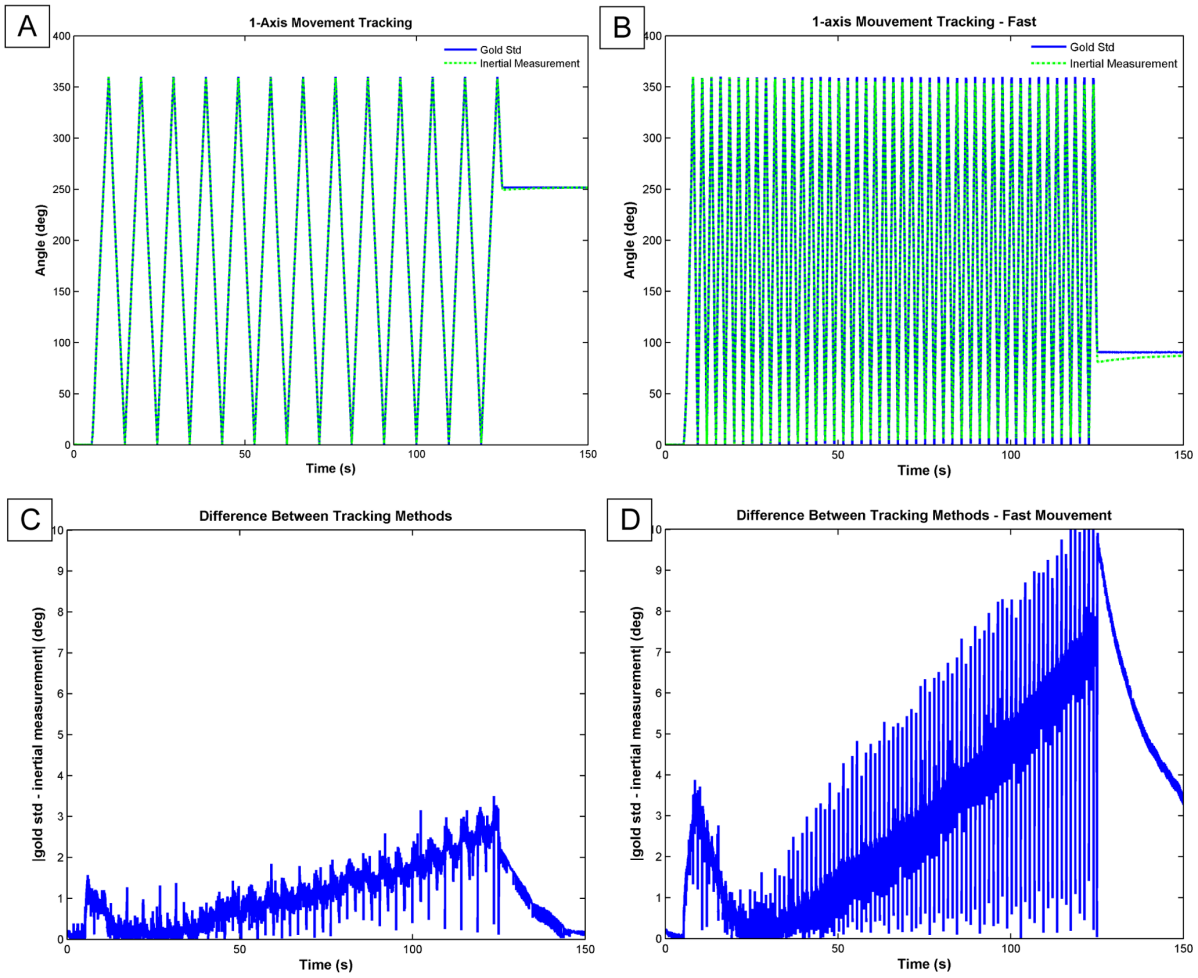
Orientation Tracking Overview. Orientation tracking with AHRS system and optical gold standard during single axis motion along with the computed error. Gimbal Table velocity at ≈90deg/s (A and C) and ≈180deg/s (B and D).

Figure 3 illustrates an overview of the motion captured by an AHRS module compared to the gold standard. The motion described in this figure corresponds to a 1-axis rotation performed at two different speeds. For slow movement (part A of the figure), both curves seem to be following each other. However, a closer look specifically at the peaks of the curves showed a slight difference between the two curves, which increased as time passed. This difference is illustrated in part C of that same figure. The right part of that same figure compares the AHRS tracking versus gold standard for that same 1-axis movement performed at higher speed. The graphic shows a more important difference between the two curves, which suggests an effect of velocity on accuracy.

### Data Reduction for Orientation Accuracy Evaluation

**Measure of accuracy.** Traditionally, movement is expressed in Euler angles as this representation is more intuitive and close to biomechanical models used to describe human motions. However, such 3D analysis strategy, just like any other 3D representation, is highly dependent upon the accuracy of the biomechanical model used to derive the proper reference frame and/or upon alignment. This study therefore proposed to directly consider the global range of motion on segmented movement, making the measurement independent of any biomechanical model. This global approach allows characterization of the AHRS modules orientation, tracking performance while removing alignment protocol uncertainties. It also does not assume a constant inertial frame throughout the movement (for comparison with gold standard).

The AHRS orientation at time *t* is therefore expressed in terms of the standardized trial’s AHRS initial orientation, using quaternions. Then, one can compare global range of motion (ROM) measured by each of the AHRS module to a gold standard. The underlying assumption regarding the equivalence of movement between the modules and the Gimbal’s center plate is considered reasonable, as the latter was designed rigidly to minimize its deformation. A quaternion is an angle-axis representation of the attitude of a rigid body; represented using a four-component vector which redundancy ensures avoidance of singularities [20]. Global ROM can be computed directly from the first component of the quaternion. From the definition of quaternion:
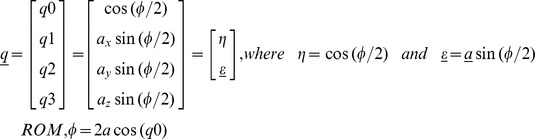



**Measure of absolute accuracy.** Absolute accuracy refers to the ability of a module to measure a change in orientation resulting from continuous movement or a change between static positions. Absolute accuracy was assessed by comparing the AHRS orientation measurement to those of a gold standard. For this study we used the Optotrak optical motion capture system from Northern Digital [21] as a gold standard. According to the manufacturer, the accuracy of the Optotrak 3020 position sensor is 0.1 mm for x,y coordinates and 0.15 mm for z coordinates at a distance of 2.25 m. The center plate of the Gimbal table was instrumented with 16 active markers from which a rigid body was built, considering a set of pre-determined constraints. These markers were tracked using 4 Optotrak camera sensors which positions were optimized so to derive the orientation of the rigid body with a worst-case precision estimated to 0.7° based on Monte Carlo analysis. Gold standard data acquisition was done through NDI First Principle v.1.2.4, at a frequency of 100 Hz. Then, AHRS data were resampled, if required, and synchronized to the reference data in post-processing using cross-correlation principal and visual confirmation. All data processing was performed in Matlab^®^ (v.7.12.0.635 (R2011a) from MathWorks).

The accuracy of AHRS was assessed by computing the mean difference in the ROM measured by the inertial system and the gold standard, hereafter referred to as 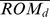. Each condition was repeated three times and measured simultaneously by four modules of the same type; hence 12 measurements are issued per condition. A Wilcoxon signed-rank test was used to evaluate the impact of velocity on the precision of the measurements, per condition. Then, following verification of the normality of the data using Kolmogorov-Smirnov test, a numerical appreciation of the impact of velocity on data precision was computed using limits of agreement [22] with a 95% confidence interval.

**Measure of relative accuracy.** AHRS modules for biomechanical applications are often used in pairs to measure relative motion at a joint. In addition to considering the ability of the involved modules to measure the same amplitude of motion when submitted to equivalent motion, relative accuracy also considers the ability of different AHRS modules to express this movement in a matching reference frame. In other words, if two AHRS modules aligned on a plane undergo the exact same movement at the same time, their relative orientation should remain the same throughout the movement. On a sensor level, one could talk about inter-sensor consistency. However, since such consistency can translate directly into a biomechanical measurement accuracy (i.e. joint angle accuracy), the authors preferred to use the term “relative accuracy”. Relative accuracy can therefore be assessed by measuring the variation of the relative orientation between modules during the different trials. The variable defined for relative accuracy is therefore the mean variation in the relative orientation for a pair of modules, hereafter referred to as 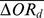. Statistical analysis strategy for relative accuracy follows the same logic as for absolute accuracy, but considering the 6 pairs of modules available for each trial, hence 18 measurements per condition.

## Results

### Absolute accuracy during static and dynamic conditions

The absolute accuracy of the measures of orientation changes between consecutive static positions was evaluated by analyzing the limits of agreement of the changes measured by the AHRS modules (4 modules per company * 4 trials  = 16 measures) to the changes measured by the gold standard using the Bland and Altman method with a 95% confidence level [22]. Overall, all three AHRS systems (Xsens MTx, APDM Opal, Inertial Labs OsV3) performed similarly in terms of absolute accuracy under static conditions. Mean differences in orientation changes measured between AHRS data and the gold standard was –0.3°±2.8° for Xsens MTx, –0.01°±2.9° for APDM Opal and –0.5°±3.3° for Inertial Labs OsV3.

**Figure 4:**
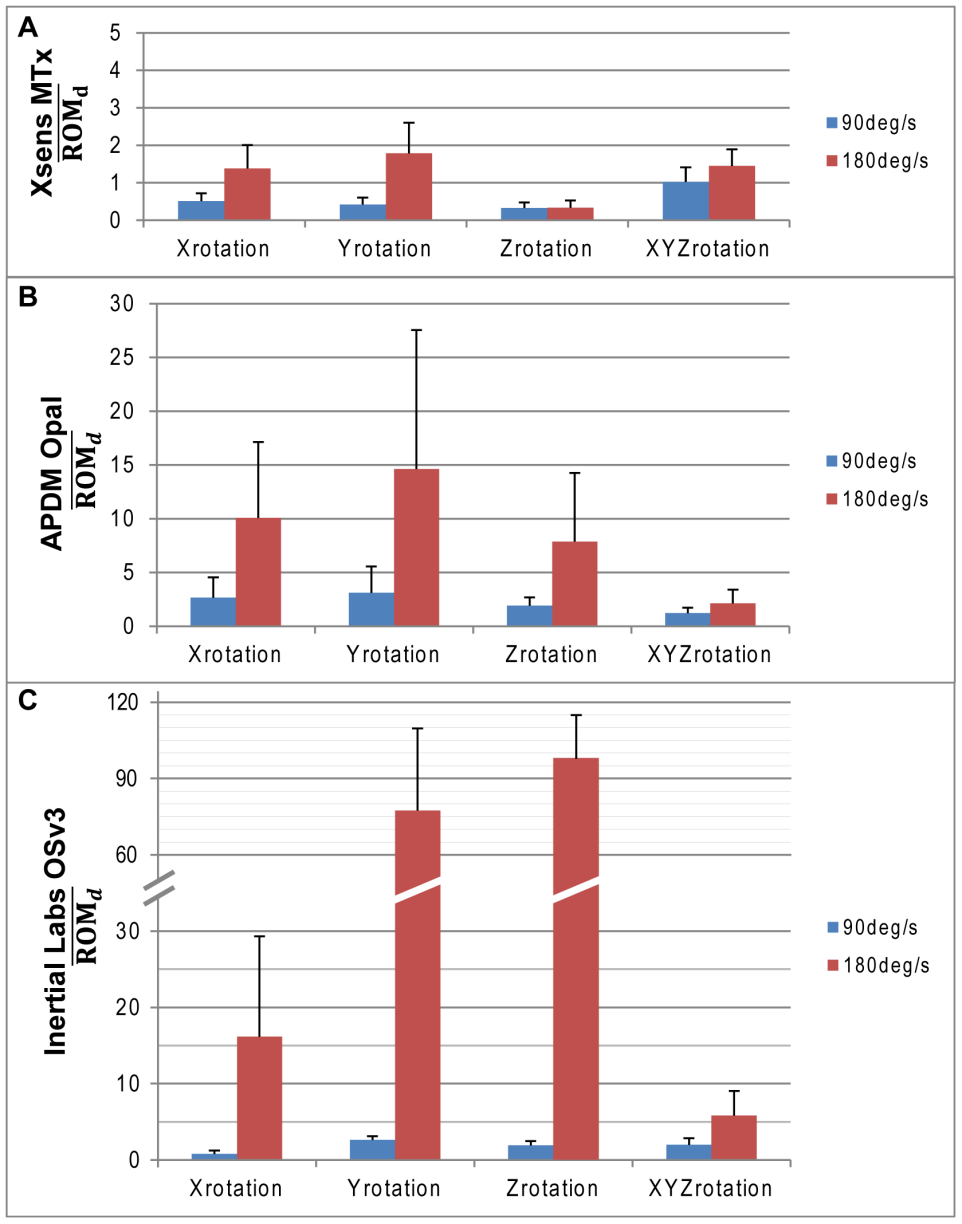
Absolute Accuracy in Dynamic Conditions. Absolute Accuracy in Dynamic Condition for (A) Xsens MTx, (B) APDM Opal and (C) Inertial Labs OSv3. ^*^Standard deviation is illustrated one-way for clarity purpose.

The differences between orientation measures for all three AHRS systems compared to the gold standard evaluated at different speeds of motion during single and multi-axis motions in the Gimbal table are illustrated in Figure 4. Averages and standard deviations are computed from twelve 30-second trials (4 AHRS modules per company * 3 trials). Under uni-axial slow motion (90°/s), differences in orientation measures between the AHRS systems and the gold standard were, on average, less than 0.5 degrees for AHRS modules from one company (0.5° around x, 0.4° around y and 0.3° around z for Xsens MTx) and slightly higher for the other two companies (0.8° around x, 2.6° around y and 1.9° around z for Inertial Labs OsV3; 2.6° around x, 3.1° around y and 1.9° around z for APDM Opal). Differences in orientation measures between the AHRS systems and the gold standard were similar for all AHRS system under multi-axial slow motions then uni-axial slow motions (1.0° for Xsens MTx; 2.0° for Inertial Labs OsV3; 1.2° for APDM Opal).

For uni-axial dynamic trials at high speed (i.e. 180^o^/s) the differences in orientation measures between the AHRS systems and the gold standard were significantly higher than those observed at slow motion. However, the impact of the speed of motion was also significantly less during multi-axis dynamic trials then single axis dynamic trials at high speed. AHRS modules from two of the three companies were incapable of tracking the orientation of the module with accuracy below 7 degrees during single axis or multi-axis motions at high speed, with AHRS modules from one company clearly diverging after few cycles (Inertial Labs OsV3).

### Relative accuracy during dynamic conditions

**Figure 5:**
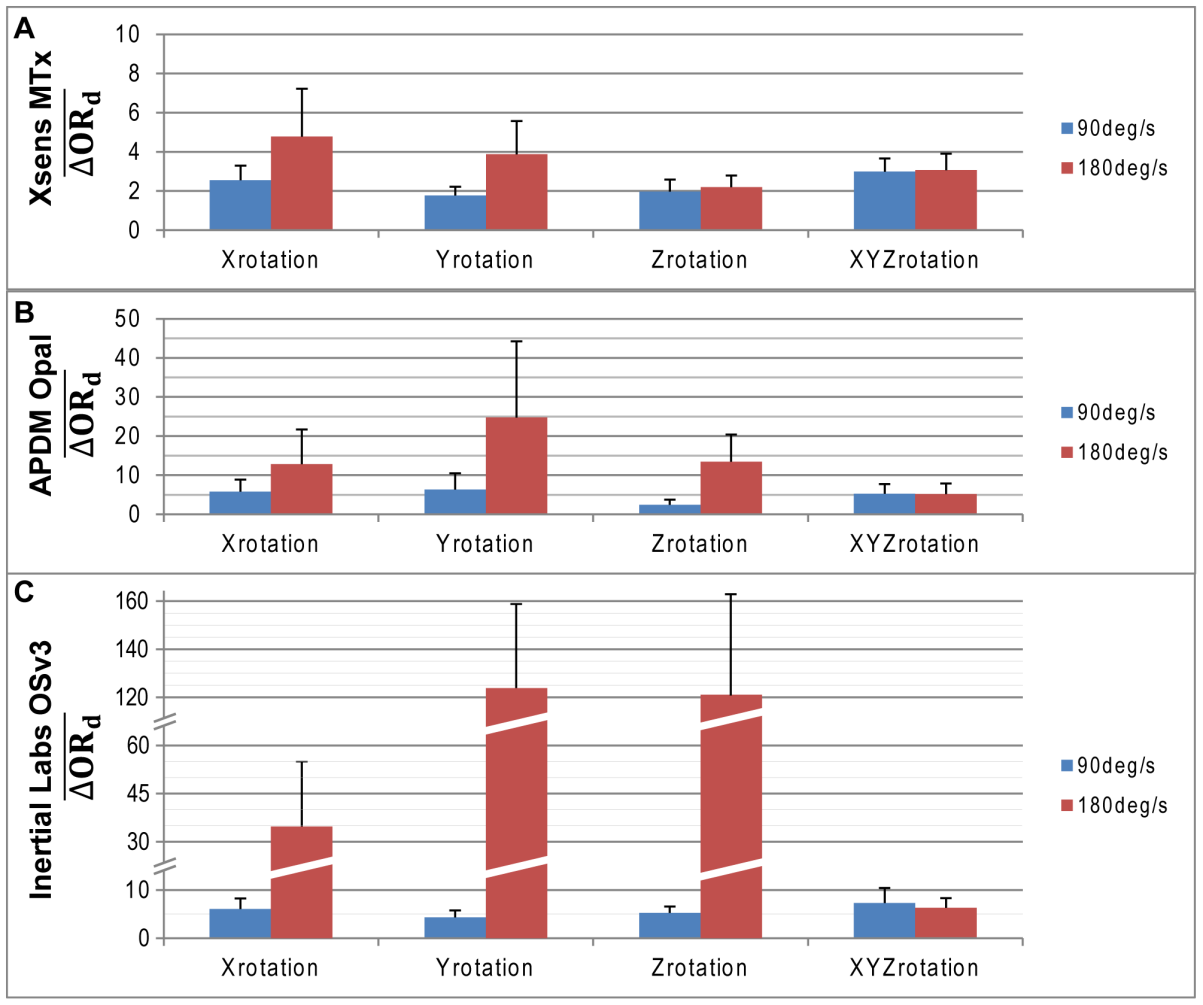
Relative Accuracy in Dynamic Conditions. Relative Accuracy in Dynamic Conditions for (A) Xsens MTx, (B) APDM Opal and (C) Inertial Labs OSv3. ^*^Standard deviation is illustrated one-way for clarity purpose.

The mean relative accuracy computed for all AHRS systems under single and multi-axis motions in the Gimbal table are illustrated in Figure 5. Results show that differences in orientation, when evaluated using pairs of modules, increases in comparison to those reported for absolute accuracy relative to gold standard under conditions of slow or fast motions (Figure 4). In slow motion, relative accuracy varied from 2 degrees to 7 degrees depending on the system and the rotation orientation (2.5° for x-rotation, 1.8° for y-rotation, 2.0° for z-rotation and 3.1 for multi-axes rotation for Xsens MTx; 5.8°, 6.3°, 2.5°, 5.3° for x, y, z and multi-axes respectively for APDM Opal; 6.1°, 4.3°, 5.3° and 7.3° for x, y, z and multi-axes rotation for Inertial Labs OsV3). Under fast motions, the best performance in mean relative accuracy was obtained with Xsens MTx modules, which have shown a precision between 2 and 5 degrees. Relative accuracy for other AHRS modules in conditions of single axis fast motions was highly variable with mean accuracy ranging between 12 degrees to 25 degrees for APDM Opal while Inertial Labs OsV3 diverged after a few cycles of single-axis fast motion. Under multi-axis conditions of motion, mean relative accuracy of all AHRS systems was less than 6.5 degrees (3.1° for Xsens MTx; 5.2° for APDM Opal; 6.3° for Inertial Labs OsV3).

### Effect of velocity

Significant differences (p<0.05, Wilcoxon signed-rank test) in absolute accuracy with respect to the gold standard were observed when comparing orientations measured under condition of slow movement versus conditions of fast movement for all but one direction of motion (Table 1). The impact of the change in velocity on absolute accuracy of AHRS modules was computed by establishing mean limits of agreement between mean differences observed under condition of slow movement’s versus conditions of fast movement for each company and each direction of movement. The mean values for the limits of agreement between conditions are listed in Table 1. The impact of changing the velocity of motions on the absolute accuracy varied across conditions and AHRS systems. Xsens MTx modules remained relatively stable with a mean difference in accuracy due to velocity below 1.4° for a cyclic movement of 30 seconds. APDM Opal AHRS modules showed differences in mean accuracy due to velocity varying from 5.9 to 11.5 degrees for single axis motions, and less than one degree for multi-axis motion. The effect of velocity for single axis motion is above 15° in the case of Inertial Labs OSv3 modules due to the observed divergence of orientation data. However, the mean difference between slow and fast mean multi-axes motion is 3.8°.

**Table 1:** Effect of Velocity on Absolute Accuracy and Relative accuracy.

	Xsens MTx		APDM Opal		Inertial Labs OSv3	
	Absolute Acc.	Relative Acc.	Absolute Acc.	Relative Acc.	Absolute Acc.	Relative Acc.
*X rotation*	*0.9°±1.2°	*2.2°±4.4°	*7.4°±10.7°	*7.0°±12.9°	*15.4°±26.7°	*28.8°±43.6°
*Y rotation*	*1.4°±1.6°	*2.1°±3.2°	*11.5°±21.4°	*18.4°±32.1°	*74.2°±65.4°	*119.4°±70.2°
*Z rotation*	0.01°±0.3°	0.2°±1.0°	*5.9°±11.6°	*11.0°±12.8°	*96.0°±34.3°	*116.0°±81.8°
*3-axes rotation*	*0.4°±0.6°	0.1°±1.6°	*0.9°±2.1°	–0.1°±4.3°	*3.8°±7.1°	–1.0°±7.0°

* Velocity effect on accuracy statistically significant with α = 0.05.

The impacts of increased velocity on the relative accuracy were greater than those observed for absolute accuracy. While Xsens MTx modules have shown a mean difference in relative accuracy remaining below 2.2° regardless of the type of motion, APDM Opals presented a mean variation varying from 0.1° to 18.4°, and Inertial Labs OSv3 modules obtained a mean difference varying between 1.0° and over 100°, again explained by the divergence of the algorithm.

## Discussion

Absolute and relative accuracy of AHRS systems are affected by environmental magnetic perturbations and conditions of motions. However, most AHRS validation studies published so far were conducted in a so-called “clean” environment where dynamic conditions were induced manually. While the latter limits trial reproducibility, the control over the magnetic environment certainly limits the inference on the accuracy conclusion to real-world conditions. This study attempts to overcome these limits with the use of a 3-axes Gimbal table commanded in velocity to enhance trial reproducibility. Furthermore, this study was conducted in a regular biomechanical lab and the different systems were configured and used according to the manufacturers’ approved protocol. Although the environmental conditions are known to be perturbed to some extent, all systems were evaluated under the same conditions for reliable bench-marking.

The AHRS systems evaluated in this study demonstrated acceptable single and multi-axis absolute angular motion accuracy under conditions of slow motion but were affected by velocity dependent errors at sustained high-speed motions. In slow motions, all three AHRS systems have shown a mean absolute accuracy below 3.1°, regardless of the direction of motion (< 1° for Xsens MTx, < 3.1° for APDM Opals; <2.6° for Inertial Labs OSv3). Xsens absolute accuracy results were within the manufacturer’s claimed values, APDM was slightly higher, by 0.3°, while Inertial Labs OSv3 technical specifications do not mention any dynamic accuracy data. Evaluation of relative accuracy, however, has shown that use of multiple AHRS modules increases the error on the measurement. Such variation in absolute versus relative accuracy tends to confirm Picerno et al. [11] statement that accuracy is partly due to the ability of all modules to locate the exact same global reference coordinate system, regardless of the environmental magnetic perturbations.

From a clinical point of view, the results of this study demonstrate that all three systems could be suitable for clinical investigation of coarse biomechanical features of motion for a given segment during slow movements (e.g. trunk inclination during transfers from siting to standing, knee range of motion during regular walk, etc…) but evaluation of more refined biomechanical features of fast segments (e.g. wrist monitoring during everyday tasks, sports biomechanics, etc.) would benefit from optimization of the fusion algorithm which relies greatly on the tuning of: (1) the fusion algorithm parameters; and (2) the magnetic compensation algorithm. Some companies address the former issue by providing different scenarios to choose from (e.g. human, human large acceleration, machine…). Although this approach is believed to help the filter’s performance, it is not available for all systems and if so, the choice of the appropriate scenario is not always obvious. For example, manufacturers recommended using the “Human” scenario for two of the systems characterized in the present study involving the Gimbal table.

The fundamentals of AHRS offer great possibilities in biomechanics although the actual way of computing relative data assumes that the magnetic environment around the modules is constant and equivalent. Current filtering practices attempt to recognize and compensate for variation in the magnetic environment relative to an “absolute” truth. However, the quality of the relative measurement does not necessarily rely on the ability of the module to locate the exact Earth’s magnetic North, but on the ability of the two modules to locate the same reference. Hence, the authors feel that a relative orientation filter could be a suitable approach. To the authors’ opinion, optimal use of AHRS system will therefore be achieved through more user-friendly approach of parameters tuning as well as more reliable magnetic compensation algorithm.

This paper also shows that velocity has a significant impact on both absolute and relative accuracy, regardless of the AHRS system considered, for almost any direction of rotation. However, the extent of this impact varies according to the system involved. A similar effect of velocity on Xsens data accuracy was also reported in [13] although the methods of evaluation, the chosen velocities as well as the duration of the trials were different. We intend to further evaluate the impact of the velocity effect on biomechanical features evaluation in clinical settings.

The Gimbal setup allows performance comparison of different systems under controlled conditions as well as systems robustness assessment. However, the Gimbal table also corresponds to extreme conditions for AHRS evaluation, as the imposed rotation is continuous while human motion can be assumed to have a zero-mean acceleration and angular speed over a certain period of time. This particularity of the Gimbal table may even explain the discrepancies observed between one-axis rotation accuracy versus multi-axes rotation accuracies. Furthermore, robustness of AHRS to such extreme conditions can also partly explain why Inertial Labs OSv3 modules diverge for high velocity 1-axis trials. Nevertheless, the Xsens MTx system has shown a greater robustness to velocity change and direction of movement than the other two systems. One possible explanation for these results is the configuration settings Xsens MTx modules allow. For the purpose of the current study, Xsens system was configured with appropriate information regarding the location of the experiment (i.e. Sherbrooke, Quebec, Canada) as well as the exact gravitational attraction corresponding to this location. These settings certainly improved the determination of the true global reference frame, enabling the correction of the angle between gravity vector and magnetic field. It is therefore believed to help the magnetic compensation algorithm. Furthermore, Xsens allows fusion algorithm tuning through the selection of pre-established scenarios (human motion, machine, etc.). These settings are believed to adjust the different algorithm tuning parameters to enhance the performance according to the type of movement and environment the modules will be used in. Inertial labs OSv3 also has some sort of scenario settings although the addition of such scenarios is fairly recent and is therefore believed to be not as tuned as Xsens’ scenarios.

## Conclusion

The main objectives of this paper were to provide an independent evaluation of market-available systems performance and to determine the ability of those systems to be used in biomechanics. The mean 3° accuracy reported in this study for slow motion allows the author to conclude that AHRS is truly an attractive solution for mobility evaluation, especially when considering their lightweight, portable and low-energy consumption characteristics. These features make AHRS modules good candidates for long-term real life mobility evaluation, although effect of time on the accuracy of the measures will need to be evaluated. The variation in the accuracy results illustrated in this paper together with the demonstrated impact of velocity on this accuracy clearly raise the importance to carefully choose the appropriate AHRS system that suits the study’s needs. However, accuracy may also relate to extensive settings or configuration procedures. Hence, when selecting an AHR system, one should clearly consider (1) the static and/or dynamic accuracy of the system, (2) the ergonomics of the system (e.g. wired vs wireless, size of modules, single-segment evaluation vs full-body kinematics…) and (3) the ease of configuration and use of the system. We have no reasons to believe that the differences in hardware configuration between the AHRS played a significant role in the differences observed in this study. So, changes in the fusion algorithm implementation and tuning could greatly improve the performance of less performing systems.

Furthermore, this study described the performance and limits of the different market-available systems under controlled conditions. However, the Gimbal table can truly be seen as a torture table for AHRS as the movement is continuous as opposed to human motion which can be assumed to have a zero-mean acceleration and angular speed over a certain period of time. For example, one can wonder what will the impact be of the bad results seen for certain systems at high speed if such high speed is in fact limited to a fraction of a second. Furthermore, the Gimbal table being fixed in the lab, the current study evaluates the performance of the systems in a single but “unclean” magnetic environment. Future work should therefore aim at evaluating the extent of the velocity effect in clinical evaluation settings as well as environmental effects, using a specific protocol designed to measure those impacts.

## References

[1] Zhou H, Hu H (2008) Human motion tracking for rehabilitation-A survey. Biomedical Signal Processing and Control 3: 1–18.

[2] Roetenberg D (2006) Inertial and magnetic sensing of human motion [Ph.D.]. Netherlands: Universiteit Twente (The Netherlands). 126–126 p. p.

[3] Cutti AG, Ferrari A, Garofalo P, Raggi M, Cappello A (2010) 'Outwalk': A protocol for clinical gait analysis based on inertial and magnetic sensors. Medical and Biological Engineering and Computing 48: 17–25. 19911214 10.1007/s11517-009-0545-x

[4] Cutti AG, Giovanardi A, Rocchi L, Davalli A, Sacchetti R (2008) Ambulatory measurement of shoulder and elbow kinematics through inertial and magnetic sensors. Medical and Biological Engineering and Computing 46: 169–178. 18087742 10.1007/s11517-007-0296-5

[5] Ferrari A, Cutti AG, Garofalo P, Raggi M, Heijboer M, et al (2010) First in vivo assessment of "outwalk": A novel protocol for clinical gait analysis based on inertial and magnetic sensors. Medical and Biological Engineering and Computing 48: 1–15. 19911215 10.1007/s11517-009-0544-y

[6] Schulze M, Calliess T, Gietzelt M, Wolf KH, Liu TH, et al (2012) Development and clinical validation of an unobtrusive ambulatory knee function monitoring system with inertial 9DoF sensors. Conference Proceedings: Annual International Conference of the IEEE Engineering in Medicine & Biology Society 2012: 1968–1971. 10.1109/EMBC.2012.6346341 23366302

[7] Giansanti D, Maccioni G, Benvenuti F, Macellari V (2007) Inertial measurement units furnish accurate trunk trajectory reconstruction of the sit-to-stand manoeuvre in healthy subjects. Medical and Biological Engineering and Computing 45: 969–976. 17653580 10.1007/s11517-007-0224-8

[8] Brodie MA, Walmsley A, Page W (2008) Dynamic accuracy of inertial measurement units during simple pendulum motion. Computer Methods in Biomechanics and Biomedical Engineering 11: 235–242. 18568821 10.1080/10255840802125526

[9] Brodie MA, Walmsley A, Page W (2008) The static accuracy and calibration of inertial measurement units for 3D orientation. Computer Methods in Biomechanics and Biomedical Engineering 11: 641–648. 18688763 10.1080/10255840802326736

[10] De Agostino M, Manzino AM, Piras M (2010) Performances comparison of different MEMS-based IMU. pp. 187–201.

[11] Picerno P, Cereatti A, Cappozzo A (2011) A spot check for assessing static orientation consistency of inertial and magnetic sensing units. Gait and Posture 33: 373–378. 21227693 10.1016/j.gaitpost.2010.12.006

[12] Brennan A, Zhang J, Deluzio K, Li Q (2011) Quantification of inertial sensor-based 3D joint angle measurement accuracy using an instrumented gimbal. Gait and Posture 34: 320–323. 21715167 10.1016/j.gaitpost.2011.05.018

[13] Cutti AG, Giovanardi A, Rocchi L, Davalli A (2006) A simple test to assess the static and dynamic accuracy of an inertial sensors system for human movement analysis. Conference proceedings : Annual International Conference of the IEEE Engineering in Medicine and Biology Society IEEE Engineering in Medicine and Biology Society Conference 1: 5912–5915. 10.1109/IEMBS.2006.260705 17946728

[14] Sessa S, Zecca M, Lin Z, Bartolomeo L, Ishii H, et al.. (2012) A Methodology for the Performance Evaluation of Inertial Measurement Units. Journal of Intelligent and Robotic Systems: Theory and Applications: 1–15.

[15] Xsens website. Available: www.xsens.com. Accessed 2013 Oct 10.

[16] Inertial Labs website. Available: http://www.inertiallabs.com. Accessed 2013 Oct 10.

[17] APDM website. Available: www.apdm.com. Accessed 2013 Oct 10.

[18] Animazoo website. Available: www.animazoo.com. Accessed 2013 Oct 10.

[19] de Vries WHK, Veeger HEJ, Baten CTM, van der Helm FCT (2009) Magnetic distortion in motion labs, implications for validating inertial magnetic sensors. Gait and Posture 29: 535–541. 19150239 10.1016/j.gaitpost.2008.12.004

[20] Hathaway AS (2009) A Primer Of Quaternions. USA: Kessinger Publishing, LLC. 136 p.

[21] Northern Digital website. Available: http://www.ndigital.com/. Accessed 2013 Oct 10.

[22] Bland JM, Altman DG (1999) Measuring agreement in method comparison studies. Statistical Methods in Medical Research 8: 135–160. 10501650 10.1177/096228029900800204

